# A180 SYSTEMATIC REVIEW OF MACHINE LEARNING-BASED PREDICTIVE MODELS FOR CLOSTRIDIOIDES DIFFICILE INFECTION

**DOI:** 10.1093/jcag/gwad061.180

**Published:** 2024-02-14

**Authors:** R Tariq, S Malik, S Khanna

**Affiliations:** Mayo Foundation for Medical Education and Research, Rochester, MN; Rochester General Health System, Rochester, NY; Mayo Foundation for Medical Education and Research, Rochester, MN

## Abstract

**Background:**

Clostridioides difficile infection (CDI) is a significant healthcare-associated infection that poses a substantial burden on patients and healthcare systems. Despite extensive research, accurately predicting CDI incidence and its associated complications remains a challenge. Electronic health records (EHRs) contain a wealth of clinical data that could potentially aid in predicting CDI and its outcomes. Machine-learning (ML) models have emerged as promising tools in healthcare, offering the potential to harness this data and enhance our ability to predict CDI incidence and complications.

**Aims:**

This systematic review aimed to evaluate the performance of machine-learning (ML) models in predicting CDI incidence and complications using clinical data from electronic health records.

**Methods:**

We conducted a comprehensive search of databases up to September 2023, adhering to the PRISMA guidelines. Studies employing ML techniques for predicting CDI or its complications were included. The primary outcome was the type of and performance of ML models, assessed using the area under the receiver operating characteristic curve (AUROC).

**Results:**

Twelve retrospective studies that evaluated CDI incidence or outcomes were included. The most commonly used ML models were random forest and Gradient Boosting. The AUROC ranged from 0.60 to 0.81 for CDI incidence, 0.59 to 0.80 for recurrence, and 0.64 to 0.88 for complication prediction. Advanced ML models demonstrated similar performance over traditional logistic regression in predicting CDI complication. However, there was notable heterogeneity in defining CDI and the different outcomes, such as incidence, recurrence, and complications, and a lack of external validation in many studies.

**Conclusions:**

ML models show promise in predicting CDI incidence and outcomes. However, the observed heterogeneity in CDI definitions and the lack of real-world validation highlight challenges in clinical implementation. Future research should focus on external validation and use of standardized definitions across studies.

**Funding Agencies:**

None

Microbiome & Microbial Therapy

